# A New Trick for a Conserved Enzyme: Mevalonate Kinase, a Glycosomal Enzyme, Can Be Secreted by *Trypanosoma cruzi* and Modulate Cell Invasion and Signaling. Is It Another Moonlighting Enzyme?

**DOI:** 10.3389/fcimb.2017.00426

**Published:** 2017-09-29

**Authors:** Diana Bahia

**Affiliations:** ^1^Departamento de Microbiologia, Imunologia e Parasitologia, Escola Paulista de Medicina, Universidade Federal de São Paulo, São Paulo, Brazil; ^2^Departamento de Biologia Geral, Instituto de Ciências Biológicas, Universidade Federal de Minas Gerais, Minas Gerais, Brazil

**Keywords:** moonlighting protein, mevalonate kinase, *Trypanosoma cruzi*, invasion, signaling

## Introduction

“Moonlighting” means doing a second job, often at night, to earn more money. So-called moonlighting proteins are proteins that play other, generally unusual and unexpected, roles in addition to their canonical roles. They have been described in a wide range of organisms from bacteria to mammals, and their additional functions vary greatly (reviewed in Collingridge et al., 2010; Huberts and van der Klei, 2010; Henderson and Martin, 2011).

The phenomenon was first described in the well-studied neuropeptides somatostatin and growth hormone-releasing factor (GRF), which were also found to have modulatory effects on the immune system. This second function was later called moonlighting (Campbell and Scanes, 1995). The term was used for the second time in a study of enzymes involved in repair of DNA double-strand breaks that were also involved in maintenance of telomeres (Weaver, 1998). However, the first study to establish the concept of moonlighting proteins was that of Jeffery (1999), who proposed that the key requirement for a protein to be classified as a moonlighting protein is that it have distinct biological functions that are not due to gene fusions, and that it be neither a multiple RNA splice variant nor a protein fragment. The additional function of a moonlighting protein may be attributed to its original active site or to novel sites (reviewed in Huberts and van der Klei, 2010; Henderson and Martin, 2011); however, in enzymes it often depends on the enzyme's catalytic activity and location. In some cases, the protein performs its original function within a cell or cell compartment and a different biological function when it is in a different cell compartment or even when it is secreted (reviewed in Collingridge et al., 2010; Huberts and van der Klei, 2010; Henderson and Martin, 2011). Hence, moonlighting activity is partially dependent on the protein's environment, a phenomenon for which the term “geographical moonlighting” has been proposed. Some moonlighting proteins have been reported to be secreted by cells and to affect intercellular signaling events, while others can act as cell surface receptors or ligands for selected receptors (reviewed in Henderson and Pockley, 2010). In eukaryotes, most proteins that display moonlighting functions are enzymes involved in metabolic processes, such as the glycolytic pathway, a universal metabolic pathway. Indeed, almost every enzyme involved in glycolysis has been reported to moonlight (Gómez-Arreaza et al., 2014), and some have been shown to play important roles in processes such as cell signaling and transformation, DNA repair, transcription, apoptosis, and detoxification, indicating that they also act in distinct subcellular compartments often unrelated to their original activity, i.e., glycolysis (Gómez-Arreaza et al., 2014; Erez and DeBerardinis, 2015). For example, Gómez-Arreaza et al. (2014) presented evidence for glycolytic enzymes (e.g., glucose-6-phosphate isomerase) in parasites that exert an unexpected second function.

## Moonlighting proteins in intracellular parasites

Numerous moonlighting enzymes normally involved in metabolic pathways in pathogenic protozoa have been reported to display different functions that enable them to act as virulence factors. For example, *Plasmodium* spp. aldolase has been reported to act as an adhesin, binding to the host cell membrane (Buscaglia et al., 2003). Aldolase has also been shown to interact with actin and surface adhesion molecules, facilitating host cell invasion by *Toxoplasma* and *Plasmodium* (Buscaglia et al., 2003; Jewett and Sibley, 2003; Baum et al., 2006).

*Plasmodium* aldolase connects motor actin filaments to proteins of the thrombospondin-related anonymous protein (TRAP) family, which are transmembrane adhesive proteins, resulting in transduction of motor energy across the parasite's surface. TRAP–aldolase interaction is crucial for host hepatocyte invasion by *Plasmodium* sporozoites and red blood cell invasion by merozoites (Buscaglia et al., 2003; Jewett and Sibley, 2003; Baum et al., 2006; Bosch et al., 2007). Interestingly, glycolytic and non-glycolytic functions of aldolases cannot occur simultaneously, because adhesins bind to the active site, blocking catalytic activity.

Enolase has been described as a multifaceted protein, and there is strong evidence that it can exert several non-glycolytic functions in pathogens (Karkowska-Kuleta and Kozik, 2014). Knowledge of the stage-specific expression and sub-cellular localization of a protein is helpful in elucidating its moonlighting functions. Enolase has also been found in the nucleus, food vacuole, cytoskeleton, and plasma membrane of *Plasmodium falciparum*, in addition to its normal location in the cytosol (Bhowmick et al., 2009).

*P. falciparum* can recruit plasminogen to the ookinete surface via the enolase internal lysine motif, DKSLVK. This process appears to be essential during degradation of the extracellular matrix (glycocalyx) that covers the mosquito midgut epithelium, because it enables the pathogen to complete a complex life cycle within its mosquito vector (reviewed in Karkowska-Kuleta and Kozik, 2014).

In the trypanosomatid *Leishmania mexicana*, enolase may be found in the cytosol and outer cell membrane as well as in the glycosome where it is normally found, and plays a part in capture of plasminogen by the parasite (Quinones et al., 2007; Vanegas et al., 2007). Hexokinase, which is generally present in glycosomes in the trypanosomatid *L. donovani*, has also been found in the flagellar pocket of the plasma membrane and displays a hemoglobin receptor-like function, suggesting that it is involved in capture of iron or heme (Krishnamurthy et al., 2005).

There are two online moonlighting protein databases: MultitaskProtDB: (wallace.uab.es/multitask) and MoonProt database (www.moonlightingproteins.org/moonlighting.php). Both contain information about moonlighting proteins for which biological evidence indicates that at least two unrelated functions are carried out by one polypeptide chain. The databases contain details for >270 proteins with confirmed moonlighting functions.

Other parasite moonlighting enzymes include *Plasmodium* dihydrofolate reductase-thymidylate synthase (DHFR-TS), which binds to its cognate mRNA and blocks its own translation, although DHFR-TS mRNA binding does not depend on enzymatic activity (Zhang and Rathod, 2002).

## *Trypanosoma cruzi* mevalonate kinase (TcMVK) as a moonlighting enzyme

Mevalonate kinase (MVK), an enzyme involved in synthesis of isoprenoids, is found in a wide range of organisms from bacteria to mammals. The mevalonate pathway is an important metabolic pathway that provides cells with bioactive molecules essential for multiple cellular processes. In the trypanosomatids *Trypanosoma brucei* and *Leishmania major*, biosynthesis of sterols is localized to multiple intracellular compartments, and HMG-CoA reductase (from acetyl CoA) and mevalonate are produced mainly in mitochondria, whereas mevalonate phosphorylation occurs only in glycosomes (Carrero-Lerida et al., 2009).

Our group characterized the ergosterol biosynthesis enzyme mevalonate kinase-TcMVK- in *T. cruzi* (Ferreira et al., 2012, 2016). This enzyme is localized in *T. cruzi* glycosomes, where it plays its conventional (conserved) role converting mevalonic acid to 5-phosphomevalonic acid (Ferreira et al., 2016). We found unexpectedly that TcMVK is also secreted into extracellular medium, where it binds HeLa cells and modulates *T. cruzi* cell invasion (Ferreira et al., 2016). Addition of a recombinant form of TcMVK to cultured HeLa cells triggered phosphorylation of MAPK pathway components and proteins involved in actin cytoskeleton changes. We further discovered that the active form of TcMVK is the dimeric oligomeric form, and only fresh purified recombinant TcMVK was capable of adding phosphate to mevalonic acid (Ferreira et al., 2016), suggesting that binding to cell host membrane and modulation of *T. cruzi* cell invasion by TcMVK are not due to its catalytic function. It has been suggested that TcMVK reaches extracellular medium by classical secretory pathways (signal peptide-mediated translocation into the endoplasmic reticulum, and secretion). Additional mechanisms such as extrusion of microvesicles may also be involved (reviewed in Watanabe Costa et al., 2016), but this possibility requires further investigation. TcMVK stimulates and regulates host cell responses to facilitate *T. cruzi* internalization, suggesting that it functions as a novel *T. cruzi* virulence factor, and is likely to have other as yet unidentified roles.

By chance, during studies of immune evasion by parasites, my attention was caught by an article entitled “Moonlighting Enzymes in Parasitic Protozoa” (Collingridge et al., 2010). I realized that TcMVK can be considered another moonlighting protein, and decided to pursue this topic. In addition to its canonical role in isoprenoid synthesis in glycosomes, TcMVK is secreted and may be involved in the modulation of cell signaling required for *T. cruzi* invasion of host cells. To date, no *Trypanosoma* enzymes have been registered in databases as moonlighting proteins. In 2016, a seven-node module (composed of nine genes) that emulates the dynamics of the parasite life cycle was identified using a systems biology approach (Carrea and Diambra, 2016). This module reproduces many important dynamic features observed in the life cycle of *T. cruzi*. In a recent Commentary, the same authors described their findings of unexpected functions in five genes that encode enzymes involved in metabolic pathways: a hexokinase, a d-1-pyrroline-5-carboxylate dehydrogenase, a quinone oxidoreductase, a glutamate dehydrogenase, and a peptidyl-prolyl *cis-trans* isomerase (Carrea and Diambra, 2017). The authors propose that these metabolic genes encode proteins with other regulatory functions distinct from their original ones, and that the enzymes should therefore be considered moonlighting proteins.

In 2015, Amblee and Jeffery performed proteomics studies that identified intracellular proteins frequently found on the cell surface, some of which displayed distinctive functions in that location and were therefore considered to be moonlighting proteins (Amblee and Jeffery, 2015). The authors suggested that these particular intracellular proteins had adopted certain biophysical features enabling them to perform a second function on the cell surface, but concluded that the moonlighting proteins had physical characteristics typical of intracellular proteins. This is also the case for TcMVK (Ferreira et al., 2016) and many other intracellular proteins that are found on the surface of pathogens and may have moonlighting activity (Gómez-Arreaza et al., 2014; Karkowska-Kuleta and Kozik, 2014).

## Moonlighting proteins and contradictory definitions

In my opinion, a precise definition of “moonlighting proteins” remains to be finalized. Several prerequisites need to be considered before deposition to the MoonProt database. In brief, a moonlighting protein is a single protein that displays multiple functions; however, the additional function(s) should be clearly demonstrated using mutagenic methods to impair the first function; i.e., the various functions should be shown to be mutually exclusive (Mani et al., 2014).

With this consideration in mind, I noticed two seemingly contradictory examples that have been deposited in MoonProt: (i) GAPDH of *Trichomonas vaginalis* was shown by (Lama et al., 2009) to perform its well-documented catalytic functions and also a second fibronectin-binding function; however, the authors did not perform mutagenic studies to demonstrate that the binding function does not depend on the catalytic residues. (ii) Nair et al. (2006) showed that hormone-induced downregulation of luteinizing hormone receptor (LHR) in human ovary granulosa cells is post-transcriptionally regulated by an mRNA binding protein, which was termed LHR-mRNA binding protein (LRBP). LRBP was subsequently shown to be identical to a MVK from rat, and to bind to the coding region of LHR mRNA, thereby suppressing its translation and resulting in degradation of the ribonucleoprotein complex. In a follow-up 2008 study of rat MVK using mutagenesis and crystallography, these authors demonstrated that Ser146, Glu193, Asp204, and Lys13 are essential for MVK binding to mRNA and also for catalytic function of the enzyme, indicating that binding of MVK to LHR mRNA in rats requires an intact active site to suppress translation (Nair et al., 2008).

In case (ii) above, the catalytic residues are necessary for the second function, which seemingly contradicts the MoonProt requisites for moonlighting proteins. On the other hand, the enzyme (MVK) had a second, unexpected function, indicating that it can be considered a moonlighting protein. The definition used for the MultitaskProtDB database is broader, i.e., moonlighting proteins are defined simply as “multitasking” proteins; however, the above proteins are not included in the database. In my opinion, both of the above-described proteins should be considered as moonlighting proteins despite the contradictory initial definitions.

In view of the inconsistency of the definitions of moonlighting proteins used for the two databases, I would like to propose a more precise definition: moonlighting proteins are proteins, typically long-known and well-studied enzymes, found to display one or more additional unexpected or unusual biological functions that were not previously anticipated on the basis of the conserved domains and-or motifs in their primary structure, or three-dimensional structure.

Our study demonstrates that TcMVK participates in parasite internalization, a process unrelated to its expected function. It can be secreted, is found on the parasite cell surface, and adheres to the host cell. Given that TcMVK adheres to the host membrane, it presumably binds to some membrane receptor to trigger yet-unidentified pathway(s) within the host.

According to Jeffery's definition (Jeffery, 1999), a protein can be classified as a moonlighting protein if it performs distinct functions depending on whether it is located inside or outside the cell, or in different cellular compartments.

I therefore suggest that the trypanosomatid protein TcMVK be considered a true moonlighting protein and be formally added to both moonlighting databases.

## Conclusion

The findings described above and the unexpected behavior of TcMVK strongly indicate that this kinase is a moonlighting protein (enzyme). On one hand, it is a conserved enzyme that is compartmentalized and plays a role in isoprenoid synthesis in glycosomes; on the other hand, it has a second, unexpected function: it is secreted, adheres to host cells, and likely participates in modulation of host cell signaling, leading to *T. cruzi* invasion of host cells. TcMVK may thus be an important modulator of *T. cruzi* invasion and a potential target in development of new drugs for treatment of Chagas' disease. This is a novel feature for a *T. cruzi* enzyme. These observations raise various questions regarding how moonlighting proteins evolved, and the selective advantages of having a single protein that performs distinctive multiple functions in an intracellular parasite.

## Author contributions

The author confirms being the sole contributor of this work and approved it for publication.

### Conflict of interest statement

The author declares that the research was conducted in the absence of any commercial or financial relationships that could be construed as a potential conflict of interest. The reviewer NR and handling Editor declared their shared affiliation.
